# The mitochondrial genome of *Sinobdella sinensis* (Synbranchiformes: Mastacembelidae) from China's Qiantang River

**DOI:** 10.1080/23802359.2021.1966338

**Published:** 2021-08-24

**Authors:** Tian-jiang Chu, Zhi Jin, Kai Liu

**Affiliations:** a Institute of Fishery Science, Hangzhou Academy of Agricultural Sciences, Hangzhou, China; b Tourism College of Zhejiang, Hangzhou, China

**Keywords:** *Sinobdella sinensis*, mitochondrial genome, next-generation sequencing, phylogeny

## Abstract

This study determined the mitochondrial genome of *Sinobdella sinensis* (Synbranchiformes: Mastacembelidae) from China's Qiantang River for the first time. The mitochondrial genome of *S. sinensis* was sequenced to be 16,543 bp in length, larger than *S. sinensis* from China's Yangtze River. The genome contains 13 protein-coding genes, 22 transfer RNAs, two ribosomal RNAs, two central non-coding regions (the control region and the origin of light strand replication), identical to most other vertebrates. Phylogenetic analysis highly supported that *S. sinensis* from China's Qiantang River was different from other *Mastacembelus* fish. However, it showed a close relationship with *Macrognathus aculeatus*. These data would explain the evolutionary mechanisms and biogeography of the family Mastacembelidae and is helpful for the conservation of genetics and stock evaluation for *S. sinensis*.

*Sinobdella sinensis* (Bleeker 1870) is an East Asian species of the family Mastacembelidae of the order Synbranchiformes, the only species in the genus *Sinobdella* according to FishBase (Froese and Pauly 2021). At present, genetic studies on *S. sinensis* are relatively few. Although the mitochondrial genome of *S. sinensis* from China's Yangtze River has been reported (Gao and Chen 2016), the mitochondrial genome of *S. sinensis* from China's Qiantang River has not been reported yet. Here, to compare *S. sinensis* from China's Qiantang River and *S. sinensis* from China's Yangtze River and phylogenetic performance among Mastacembelidae fish, the mitochondrial genome of *S. sinensis* from China's Qiantang River was determined.

*S. sinensis* was harvested from the Qu River, an upstream tributary of China's Qiantang River (119°04′1.326″ E, 29°00′40.453″ N), and deposited at the National Original Breeding Farm of black Amur bream from China's Qiantang River (120°07′21.99″E, 30°08′35.53″N). The total genomic DNA from the fin tissue (assigned as ZHCQ202103) was extracted by the phenol–chloroform extraction method (Green and Sambrook 2012). After the genomic DNA was quantified, the DNA was sonicated using a Covaris M220. The sheared DNA fragments were purified and used to construct a sequencing library and subjected to next-generation sequencing (NGS). The NGS was performed by Origingene Bio-pharm Technology Co., Ltd. (Shanghai, China). The mitochondrial genome of *S. sinensis* was obtained by sequence assembly on the NGS data using GetOrganelle ver. 1.7.3.5 (Jin et al. 2020). The accession was registered GenBank under accession numbers MZ188892. The annotation process was completed using the MITOFISH prediction server (Iwasaki et al. 2013).

The complete mitochondrial genome of *S. sinensis* from China's Qiantang River is 16,543 bp, larger than *S. sinensis* from China's Yangtze River (Gao and Chen 2016) due to the 12S rRNA out of three nucleotides. The mitochondrial genome contains 22 tRNAs, two ribosomal RNAs, 13 protein-coding genes (PCGs), and two central non-coding regions. Most genes of *S. sinensis* are encoded on the heavy strand (H-strand) except for *ND*6 and eight tRNA, which are encoded on the light strand (L-strand). Within the genome, all the 13 PCGs included the orthodox start codon ATG except for *CO*1, which is initiated with GTG. However, the stop codons of the 13 PCGs differ, these terminating with TAG, TAA, TA–, or T–. The origin of light strand replication (OL), which extends up to 31 nucleotides, is identified in the WANCY region. The second non-coding region, the control region (D-loop), is located between the tRNA-Pro and tRNA-Phe with 919 bp in length. The phylogenetic tree of *S. sinensis* is shown in Figure 1, drawn by IQ-TREE ver. 2.1.3 with the complete mitochondrial genomes, under the TIM2 + F + G4 substitution model (Minh et al. 2020). The phylogenetic tree clearly shows that *S. sinensis* from China's Qiantang River is different from other Mastacembelus fish. However, *S. sinensis* from China's Qiantang River is not initially grouped with *S. sinensis* from China's Yangtze River but clustered first with *Macrognathus aculeatus* from China's Pearl River. SH-aLRT is a fast nonparametric version of an approximate likelihood ratio test (aLRT) developed and implemented in the PHYML phylogenetic inference software (Anisimova et al. 2011). The SH-aLRT test values on the nodes of *S. sinensis* MZ188892, *M. aculeatus* KT443991, and *M. aculeatus* KF636363 are higher than 80%, which means the credibility of the SH-aLRT test is high (Hoang et al. 2018). However, the Ultrafast Bootstrap values on the nodes are low. The phylogenetic relationship still needs more attention between *Macrognathus* spp. and *Sinobdella* spp., although there are many similarities in morphology.

**Figure 1.**
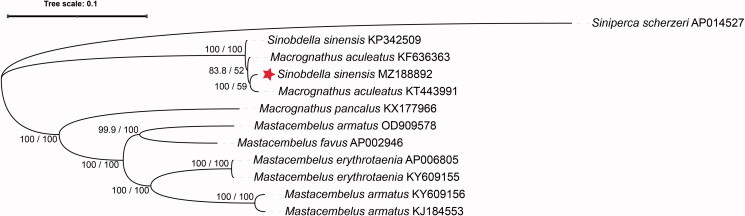
Phylogenetic tree of *Sinobdella sinensis* inferred using the maximum-likelihood method based on the mitochondrial genomes. Values are shown at each node of the tree, correspond to the SH-aLRT test values and Ultrafast Bootstrap value given in percentages.

## Data Availability

The data that support the findings of this study are openly available in National Center for Biotechnology Information (NCBI) at https://www.ncbi.nlm.nih.gov/nuccore and https://www.ncbi.nlm.nih.gov/sra, reference number MZ188892 and PRJNA737155.
